# A CHALLENGE HONORING SELF-DETERMINATION OF NURSING HOME RESIDENTS WITH DEMENTIA; WHICH SELF -- THEN SELF OR NOW SELF?

**DOI:** 10.1093/geroni/igae098.3773

**Published:** 2024-12-31

**Authors:** Mercedes Bern-Klug, Meredith Levine

**Affiliations:** University of Iowa, Iowa City, Iowa, United States; The Harry and Jeanette Weinberg Center for Elder Justice at The Hebrew Home at Riverdale, Bronx, New York, United States

## Abstract

Medicare and/or Medicaid certified nursing homes (NHs) are required to inform residents they have a right to an advance directive. The government’s Resident Rights requires staff to honor resident self-determination. What happens when instructions in an advance directive are in opposition to the current expressed requests of a person with advanced dementia? Using qualitative descriptive methodology, in this pilot study, 12 NH staff members in various roles engaged in individual interviews and responded to questions that speculated they had a resident with dementia who, when they had decisional capacity, indicated in a written advance directive they did not want to be fed, once they lost the ability to feed themselves. Staff were unanimous in declaring the need to honor the resident’s self-determination, and yet could foresee instances in which a person with said advance directive, and unable to self-feed, would indicate through words or gestures, the desire to eat. Many staff indicated they would go ahead and feed the person; others worried that would dishonor the advance directive. Staff also expressed other challenges including how “self-feed” is operationalized, the fluctuating nature of functional capacity in dementia, and the potential conflict that could result with families and NH surveyors. Some staff indicated it would be against their religion or sense of morality to not feed a person who expressed an interest in eating. We conclude that although such advance directive templates are readily available online, the potential for logistical and moral challenges in the NH context are real and serious.

